# Supplementary figures and images for: Mouse papillomavirus type 1 (MmuPV1) DNA is frequently integrated in benign tumors by microhomology-mediated end-joining
Source: PLoS Pathog. 2021 Aug 3;17(8):e1009812. doi: 10.1371/journal.ppat.1009812 (PMC8362953; doi:10.1371/journal.ppat.1009812)

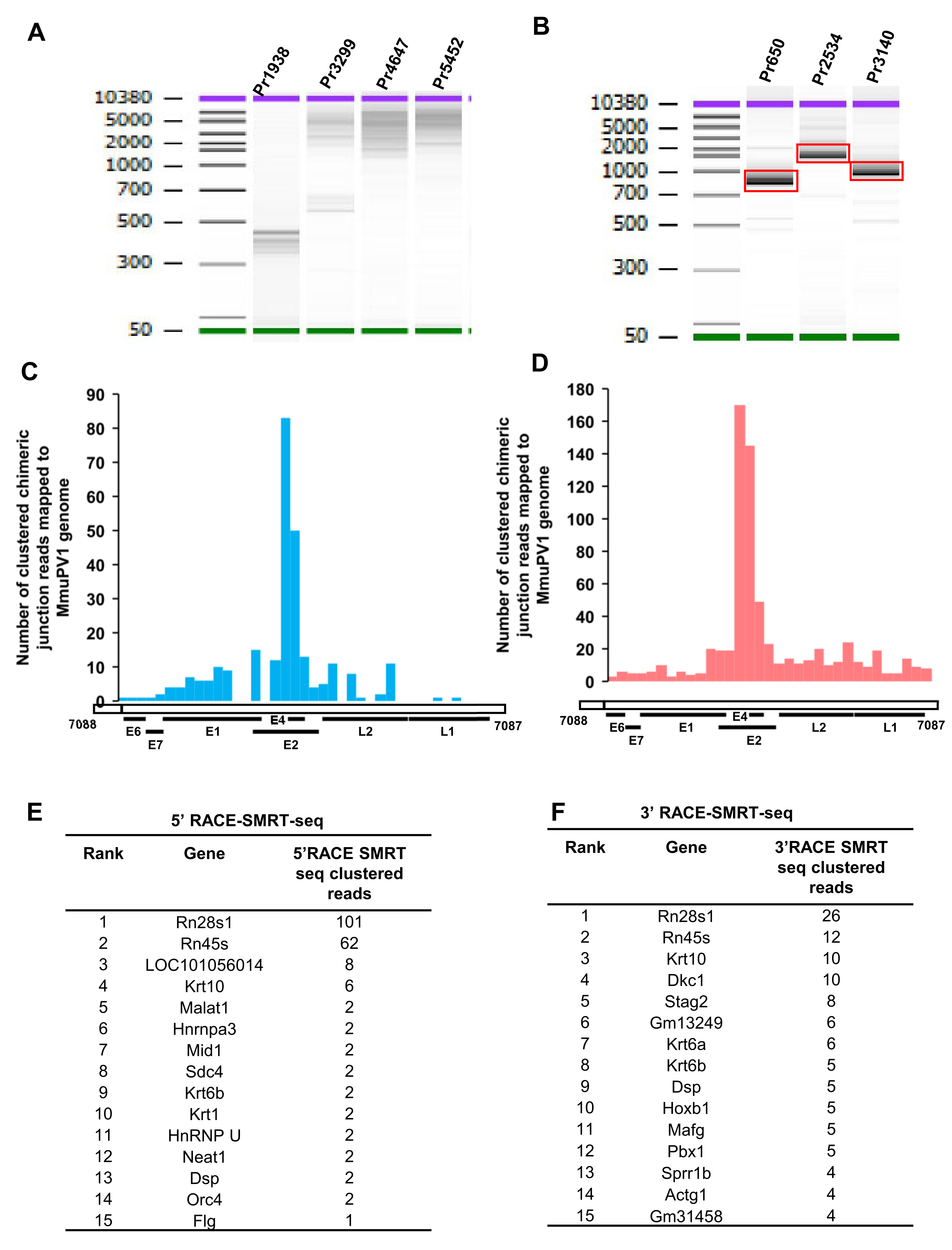

Supplement: S1 Fig — A and B, 5′ RACE products (A) and 3′ RACE products (B) of MmuPV1 transcripts were identified using different MmuPV1-specific primers on total RNA isolated from MmuPV1-induced tumor tissues. All the products from 5′ RACE were analyzed by SMRT-seq to identify chimeric virus-host RNA transcripts (A). After removing the major viral polyadenylation products derived from transcription of MmuPV1 episomal DNA (red rectangles), the remaining 3′ RACE products (B) were analyzed by SMRT-seq for chimeric virus-host RNA transcripts. C and D, Distribution of integration breakpoints across the MmuPV1 genome identified by 5′ RACE (C)- and 3′ RACE (D)-SMRT-seq. E and F, Top10 host genes with MmuPV1 integrated DNA as detected by 5′ RACE (E)- and 3′ RACE (F)-SMRT-seq. (TIF) [file ppat.1009812.s001.tif]

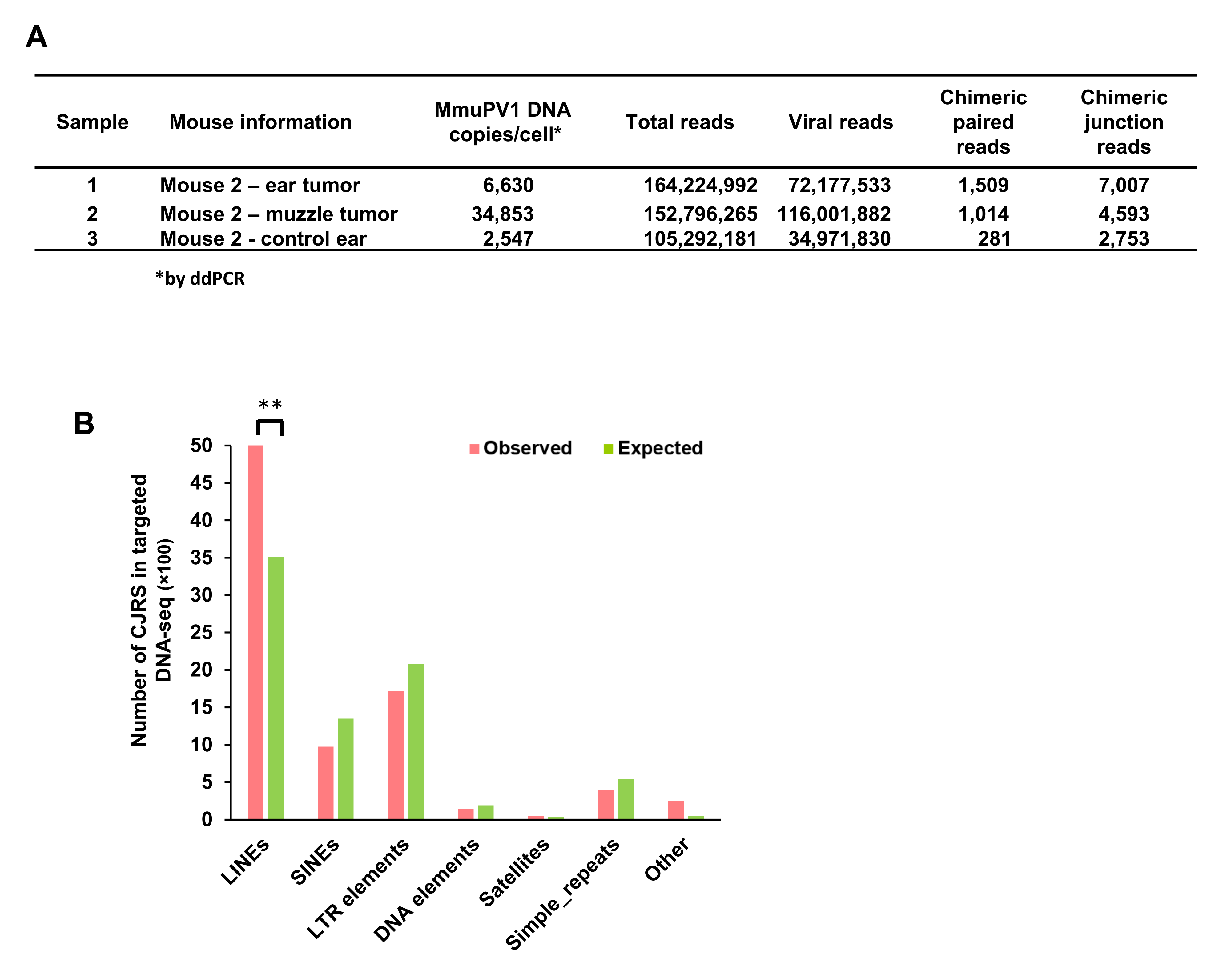

Supplement: S2 Fig — A, DNA CJRs from MmuPV1 tumor and non-tumor (control) tissues were identified by targeted DNA-seq. MmuPV1 copy number per cell was determined by ddPCR from 10 ng of genomic DNA using the mouse Tfrc gene as an internal control. B, Expected and observed CJRs in different interspersed repeats in the mouse genome identified by targeted DNA-seq. Repeat data for the mm10 genome was downloaded from repeatmasker.org website (Repeat Library 20140131). "intersectBed" command from bedtools (https://doi.org/10.1093/bioinformatics/btq033) package was used to identify the virus-host junctions that mapped to known repeat regions. **, P<0.01 by a chi-squared test. (TIF) [file ppat.1009812.s002.tif]

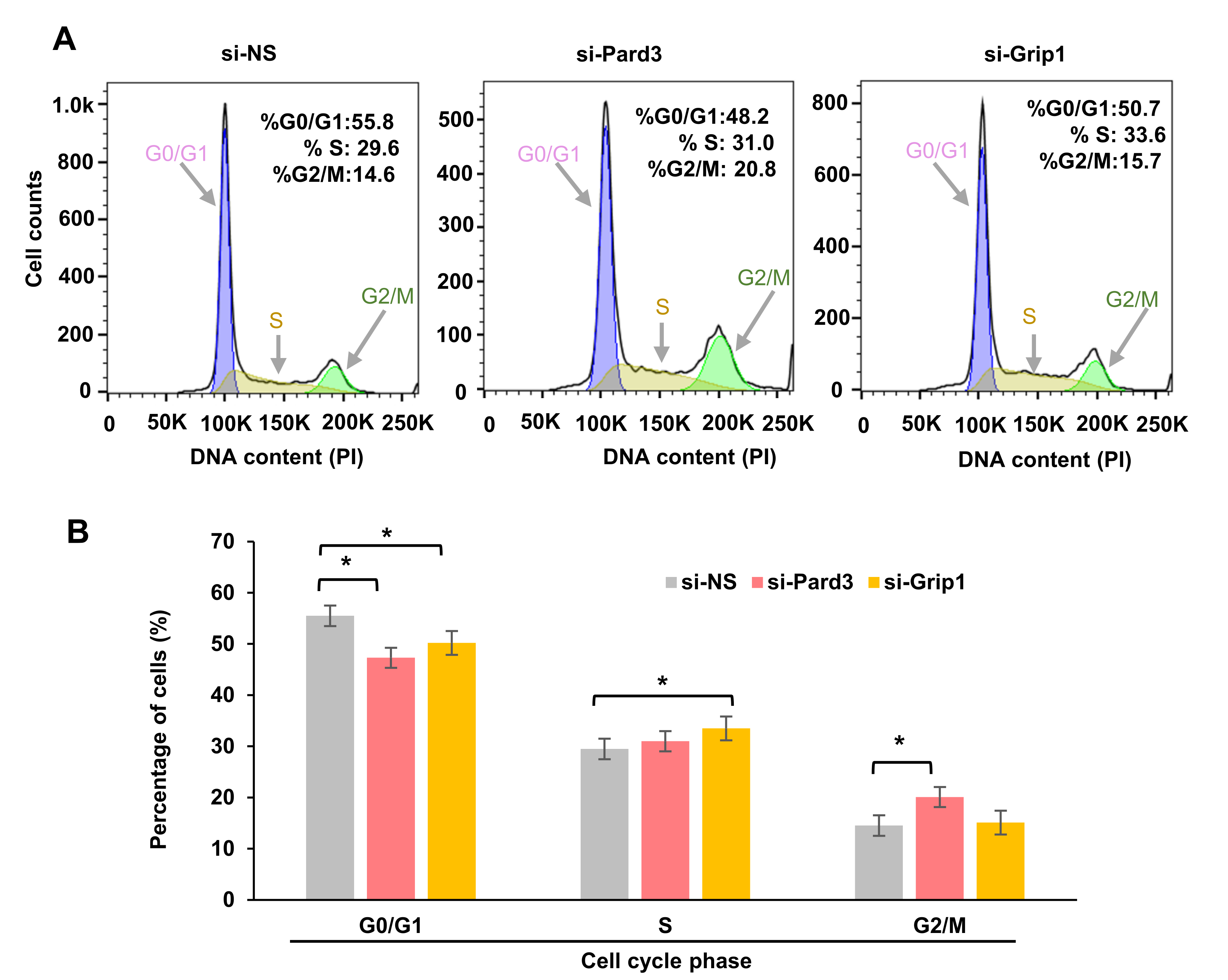

Supplement: S3 Fig — A, Flow cytometry analysis of mouse keratinocytes with reduced expression of Pard3 and Grip1 after gene-specific siRNA treatment. The mouse primary keratinocytes were transfected twice with 40 nM of siRNA (si-Pard3, Si-Grip1 or si-NS) at a 24 h interval. Cells were fixed 24 h after the second siRNA knockdown (KD) and analyzed by flow cytometry in triplicate. PI, propidium iodide. B, Bar graphs show the cell cycle distribution after two rounds of siRNA KD of Pard3 and Grip1 expression. Data are the mean ± SD (n = 3). * P<0.05 by paired, two-tailed Student’s t test. (TIF) [file ppat.1009812.s003.tif]
