# Supplementary material for: Mouse papillomavirus type 1 (MmuPV1) DNA is frequently integrated in benign tumors by microhomology-mediated end-joining
Source: PLoS Pathog. 2021 Aug 3;17(8):e1009812. doi: 10.1371/journal.ppat.1009812 (PMC8362953; doi:10.1371/journal.ppat.1009812)
Supplement: Attachment — Submitted filename: Response to Reviewers-LLY.docx [file ppat.1009812.s014.docx]

**Our responses to all reviewers**

**Reviewer #1:** Manuscript: PPATHOGENS-D-21-00964 Title: Mouse papillomavirus type 1 (MmuPV1) DNA is frequently integrated in benign tumors

by microhomology-mediated end-joining.

Authors: Lulu Yu, Vladimir Majerciak, Xiang-Yang Xue, Aayushi Uberoi, Alexei Lobanov, Xiongfong Chen, Maggie Cam, Stephen H. Hughes, Paul F. Lambert, and Zhi-Ming Zheng.

This manuscript describes novel findings in the MmuPV1 mouse infection model to determine whether this virus infection can lead to viral integration that would lead to malignant progression, and further delineate additional mechanisms to explain the observations that MmuPV-1 infections can progress to malignancy. At present, the MmuPV1 model has shown clear evidence for malignant progression, but much of the current literature has focused on early infections and a mechanism for the malignant phenotype is lacking. The current study provides a comprehensive and mechanistic model for the potential for viral integration by demonstrating that even in the early stages of benign infections, there is evidence of viral transcripts that are spliced to host genetic elements. These events would increase the statistical likelihood that viral integration is possible in this model and that MmuPV1 could be categorized as a member of the PVs that demonstrate malignant potential in the natural host.

The study is a powerful and comprehensive documentation of these rare but potentially clinically significant integration events and set the stage for more in-depth analyses of HPV infections from more benign HPV types.

Thanks for the positive comments on our comprehensive analyses of MmuPV1 integration in MmuPV1-induced mouse tumors.

Some items for consideration:

1. The Abstract has several abbreviations that would benefit from expansion (or a list of abbreviations if not already in place).

Thanks for your comments. We have spelled out the RACE (rapid amplification of cDNA ends and SMRT (Single-molecule real-time) seq in the abstract (line 28, 29) for better understanding.

2. The observation that integration events are observed early in infection is particularly novel as histological and current virological assays would identify MmuPV1 early infections as benign and that most viral DNA are episomal. Intriguingly, these studies also indicate that such relatively rare integration events are ongoing during the disease progression and are a novel explanation as to how HPV integration can lead to cancer (increased survival advantage of rare cells with integrated events). More importantly, such events may be ongoing in infections with other HPV types that are less carcinogenic, and this could also set the stage for cancer development due to the ongoing host-cell “insults”. These findings are worthy of a future in depth study of HPV lesions from more benign HPV types (particularly the various skin HPV types) that are suggested to be a co-factor in non-melanoma skin cancer despite the difficulty of detecting integrated HPV DNA in tumor tissues.

We are grateful for your positive comments on our study. We hope our novel observations from this mouse model will provide an exciting direction for understanding HPV integration-induced progression of carcinogenesis.

**Reviewer #2:** MmuPV1 serves as a valuable mouse model for papillomavirus infection in both immunocompromised and immunocompetent animals. This paper reports integration sites/frequency after experimental MmuPV1 infection of BALB/c FoxN1nu/nu mice (immunodeficient). Mice were typically infected for 6M to allow wart formation. Using RNA-seq, RACE-SMRT-seq, and/or targeted DNA-seq they describe integration, consistent with a Microhomology-mediated end-joining (MMEJ) mechanism. They provide interesting correlative data regarding increased integration and infection/neoplasia and host MMEJ factors that are increased following infection/integration, and speculate (with good discussion) regarding the mechanisms behind the observed phenomena. Integration often resulted in disruption of E2, which would be expected to cause a selective advantage of those integrants if they integrate near a usable host polyadenylation signal to enable expression of early viral genes.

The science is sound and the paper is well written and clear. The work is important, laying the ground for testable hypothesis moving forward, and very appropriate for this journal.

Thank you for the positive comments on our comprehensive analyses of MmuPV1 integration in MmuPV1-induced mouse tumors.

A few concerns/issues are outlined below, which if addressed would strengthen the paper in my opinion.

Concerns/Issues:

1. The data in Figure 7. While the data show modest upregulation of these factors at the transcriptional level, their effects would be exerted at the protein level. Validation of this data by western blot would strengthen the conclusions from this data.

Thanks. Accordingly, we have validated the expression of increased FEN1 protein and phosphorylated (S139) H2AX protein (γH2AX) by Western blot. The new data are included in the revised Figure 7C and described in the text (lines 566-569).

2. Figure 2b- the table indicates that there were ~1.3M viral reads for the mouse 1 infected ear, and ~972K viral reads for the control ear (uninfected) from the same animal. This seems like way too many viral reads from an uninfected control ear, can this be explained? Was there transmission from primary infections to these "uninfected" sites during those 6M? If so why weren't there any warts in these "uninfected" sites? (or were there?). Did any warts appear in uninfected sites during the experimental infections?

Thank you for the comment. The RNA-seq analysis of three control ears without virus inoculation revealed many viral reads (29K~971K) in these control tissues. However, there were no visible warts, yet there was clearly infection also based upon RNA scope (data not shown). The simplest explanation is that there was lateral transmission occurring between experimentally infected ears and experimentally uninfected ears in these immunodeficient mice. There are other possible reasons for the infection spread to the control ears that did not cause tumor formation. As we published, first, MmuPV1 and CRPV could be transmitted to other sites through blood (Cladel NM et al, Emerg Microbes Infect. 2019. doi: 10.1080/22221751.2019.1637072). Second, tissue pre-wounding is essential for skin wart induction as demonstrated for CRPV and MmuPV1 infection. Thus, it is understandable that control ear has virus infection, but no wart formation.

3. Line 389-390: "There were multiple integrations in Malat1, Flg, Krt1, Dsp, Krt10, Hmr and Rn7sk (Fig. 2E, Supplementary Table S2)." Are these the only genes with multiple integrations from this data? Table S2 seems to suggest many genes had multiple integrations, unless I'm confused?

Thanks! You are right. These genes are not the only genes with multiple integrations as you can see from the Table S2. We listed the top integrated genes with multiple integration as examples in Fig. 2E.

4. Line 462-463: "although the DNA CJRs from these genes did not have the same junction sequences that were identified in the RNA CRJs." Why is this? Additional discussion about these discrepancies is needed.

RNA-seq detects only the expressible integrant and the RNA reads from the exons or unspliced (rare) regions. Because majority of the genome are noncoding regions, the detected DNA CJRs (~93%) in our study were found, as expected, predominantly from the noncoding intergenic and intronic regions of the mouse genome, where they are either inexpressible or spliced out if they are expressed as a part of the intron of coding genes. This is the main reason why the RNA CJRs is different from DNA CJRs. We had this description at the beginning of the paragraph in our previous submission. To avoid confusion, we deleted this sentence in this revision.

5. Similar to #2 above, RNA-seq library prep can lead to sequencing artifacts (as recently highlighted with the whole SARS-CoV-2 integration debacle, and papers like PMID 33980601 refuting that shoddy work). This work would be improved with a thorough discussion of these technical issues, and what was done here to prevent or recognize artifactual sequencing data.

Thanks for the good point! We totally agree with this critical comment. That was the reason why we were not dependent on a single method in our study, but rather the data have to be verified as designed in this report by multiple technics. For both RNA-seq and targeted DNA-seq pair-end read integration analysis, we applied very stringent criteria to identify the chimeric reads, of which the identified chimeric junction read (CJR) has at least 20 nts being aligned to the both MmuPV1 and mouse genomes on each side of the junction. As shown in the last paragraph of introduction part, SMRT-seq (single-molecule sequencing technology or PacBio, Pacific Biosciences) that produces long reads (up to >50 kb) was also used to further verify the RNA-seq results (Fig.3). Additional RT-PCRs were applied to selectively verify the RNA-seq junction (Fig.6D).

6. Figure 1A- please indicate/confirm this was a 6m infection in the text, figure and/or legend. It is unclear from reading the results text on line 352.

Has been done accordingly in the Fig. 1A legend (line 959-960).

7. Are homologous sequences in the range of 2-3 nt really long enough to be classified as "microhomology"? These seem too short. Perhaps a comment about this or some citation of literature discussing this would improve. If the 2/3 nt data are removed does it affect the conclusions?

Microhomology-mediated end-joining (MMEJ) is a double stand break repair mechanism that uses short regions of homologous DNA sequences (>=1bp) for error-prone end-joining (line 92). A single-nucleotide homology has been found in DNA repair even in C. elegans to allow DNA polymerase Theta acting on and extending a one-base-pair intermediate in an alternative end-joining repair mechanism (Koole, W, et al. Nat. Commun. 5: 3216, 2014). As suggested by the reviewer, we cites this reference and other two review articles in line 91 in the introduction section. To be safe, we included >=2nt homologous sequence for our microhomology analysis. We did the statistical analysis using different length of microhomology sequence in both RNA-seq (Fig 6A, 2 -12 nt) and DNA-seq (Fig 6B, 2-10 nt), and showed all of them being significantly enriched in tumor tissues over the control tissues. Therefore, removing the 2-3 nt data will not affect our conclusions.

**Reviewer #3:** Mouse papillomavirus type 1 (MmuPV1) DNA is frequently integrated in benign tumors by microhomology-mediated end-joining

The authors investigated integration of the murine papillomavirus MmuPV1 into the genome of

BALB/c FoxN1nu/nu mice at three skin sites known to be susceptible to MmuPV1-induced papilloma formation (tail, muzzle, ear). They provide evidence that the viral DNA is integrated in infected cells, particularly in benign skin papillomas. This topic is of utmost interest to the papillomavirus field and the findings presented herein are novel. The manuscript is well written, contain important data and the experiments are sound and well performed.

Many thanks to this reviewer for his/her positive comments on our work!

1. By RNA-seq analysis the proportion of virus-host chimeric reads were much higher in n=9 MmuPV1-induced tumor tissues (1.9-7) compared to n=3 MmuPV-1-infected tumor-free skin (0.6-1.3). Are the results statistically significant?

Thank you for the comment. The proportion of virus-host chimeric reads in MmuPV1-induced tumor tissues (1.9‰ - 7‰) is significantly higher than that in MmuPV-1-infected tumor-free skin (0.6‰- 1.3‰), with p<0.01 by student t-test. See revised Fig. 2B.

2. The numbers of integrated mucosal high-risk HPV copies correlate with severity of cervical disease and progression. MmuPV1 infection led to development of SCCs in ear skin (ref 39). Did the authors have a chance to look into integration sites in MmuPV1-induced SCCs or their premalignant precursors on ear skin? Do they think that higher levels of integrated virus may correlate with increasing malignancy in virus-induced skin cancer, similar to the cervix?

Yes, our collaborator Dr. Paul Lambert’s group is working toward this direction although we did not do so in this report. Nevertheless, we demonstrated that the integrated chimeric reads in tumor tissues were much higher than the infected, tumor-free tissues (Fig. 2B) and in a step-wise increase in the expression of the DNA repair MMEJ host factors from normal tissues, to tumor-free MmuPV1 infected tissues, and then to MmuPV1 tumors (Supplemental Table S10).

3. When did the authors harvest the papillomas, the latest 6 months post-infection? As immunodeficient FoxN1 nude mice were used, it is unlikely that the papillomas will regress. Do the authors expect more (new) genes affected by integration in the aging mouse, e.g genes responsible for cancer growth and development, after 1 or 2 years.

Yes, we harvested the papillomas at 6 months post-infection (line 129). Unfortunately, our study did not include any mouse tumor from the mouse aged longer than 6 months after virus inoculation. Thus, we are unable to address this interesting question. Theoretically, more host genes would be affected in an aged mouse with MmuPV1 infection.

4. Could the authors please state the number of animals analyzed in the Material and Methods section.

Sorry for the confusion. Detailed mouse information was shown in Fig. 2B in our previous submission. As requested, we also added this information in Material and Methods (line 125-129) in this revision.
